# Acute Marjolin's Ulcer in a Postauricular Scar after Mastoidectomy

**DOI:** 10.1155/2016/2046954

**Published:** 2016-12-06

**Authors:** Kholoud A. Alhysoni, Sumaiyah M. Bukhari, Mutawakel F. Hajjaj

**Affiliations:** Otolaryngology Department, Ohud Hospital, Medina, Saudi Arabia

## Abstract

*Background*. Marjolin's ulcer is a rare, aggressive cutaneous malignancy that arises primarily in burn scars but can occur in other types of scars. Squamous cell carcinoma is the most common variant, and while malignant degeneration usually takes a long time, it can develop acutely.* Case Report*. a 30-year-old man who developed Marjolin's ulcer acutely in a right postauricular scar after mastoidectomy and the incision and drainage of a mastoid abscess. To the best of our knowledge, this report is the first to describe a Marjolin's ulcer in a postauricular surgical scar. However, it has been reported in others areas in the head and neck.* Conclusion*. Marjolin's ulcer is most commonly observed after postburn scars, but it may be observed after any type of scars, as our patient developed an SCC with a postsurgical scar. Early diagnosis is essential, and a biopsy should be performed on any nonhealing wound or chronic wound that undergoes a sudden change. Tissue samples should be taken from both the centre and the margins of the wound.

## 1. Introduction

Marjolin's ulcer refers to cancer that most often presents in an area with a chronic burn wound. Marjolin's ulcer is also associated with nonhealing wounds, venous ulcers, lupus vulgaris, vaccination scars, snake bite scars, chronic osteomyelitis fistulae [1], amputation stumps, cystostomy sites, chronic lymphedema, chronic pilonidal sinuses [2], pressure ulcers in spina bifida patients [3], ischial bursitis [4], hidradenitis suppurativa [5], posttraumatic scars [6–9], surgical scars [10], and scars after coronary artery bypass grafting [11].

The most commonly affected sites are the lower extremities, followed by the head and neck region and the trunk [1, 11]. The most commonly involved areas of the head are the scalp [1, 6] and face [1, 10]; in one reported case, the nose was affected [12], and in another, the neck was affected [8].

We report the case of a 30-year-old man who developed Marjolin's ulcer in the right postauricular area only 9 months after the incision and drainage of a right mastoid abscess. There are no other reports of Marjolin's ulcers in this area to date.

## 2. Case Presentation

A 30-year-old Bangladeshi male presented to the emergency room with a five-day history of right postauricular swelling that had gradually increased in size. There was associated fever and purulent discharge from the right ear.

The patient had a longstanding history of right ear discharge and decreased hearing in the right ear with no tinnitus or vertigo.

The patient had no medical illness and was negative for human immunodeficiency virus.

Examination revealed a right mastoid swelling that was fluctuant, hyperemic, tender, and warm. Needle aspiration revealed 6 cc of purulent fluid. The right external auditory canal (EAC) and right tympanic membrane perforation emitted purulent discharge. The facial nerve was intact on examination, with no palpable lymph nodes.

Pure tone audiometry showed right profound mixed hearing loss.

**Figure 1:**
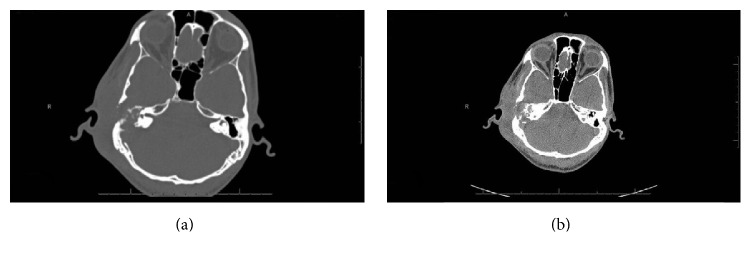
Axial CT scan (noncontrast) showing the temporal bone. (a) Bone window and right ill-defined soft tissue density in the right mastoid air cells, middle, and inner ear, associated with bony destruction. Only the basal turn of the cochlea and part of the vestibule are visualized. (b) Soft tissue window and posterior aspect of the tegmentum tympani appear destroyed, thinned, and interrupted, with subcutaneous soft tissue swelling adjacent to the EAC and collection at the superior aspect.

Axial computerized tomography of the temporal bone showed a mastoid abscess with bone destruction (Figure 1).

**Figure 2:**
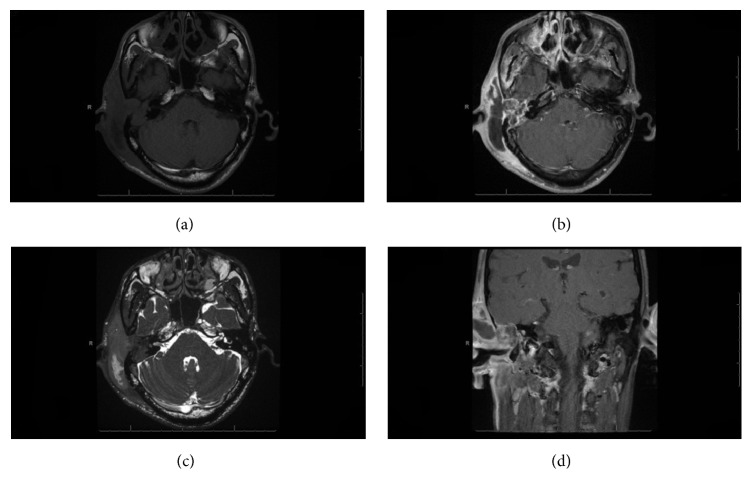
MRI of the brain, IAC, and mastoid with IV contrast. Axial and coronal views show (a) MRI T1 axial view before contrast, (b, c) MRI T1 axial view after contrast, and (d) MRI T1 coronal view after contrast. Right, large, loculated, peripheral enhancing collection is observed in the subcutaneous tissue of the auricular region, posterior and anterosuperior to the external auditory canal and extending to the mastoid air cells and middle ear cavity. The cochlea and semicircular canals are not visualized; only part of the vestibule is observed, and a focal area of dural enhancement is observed in the right temporal lobe.

Magnetic resonance imaging of the brain showed enhanced collection in the subcutaneous tissue and auricular region posterior and anterosuperior to the EAC that extended to the mastoid cavity and the middle ear cleft. There was focal area of dural enhancement observed in the right temporal lobe (Figure 2).

A diagnosis of right chronic suppurative otitis media complicated by mastoid abscess was made.

**Figure 3:**
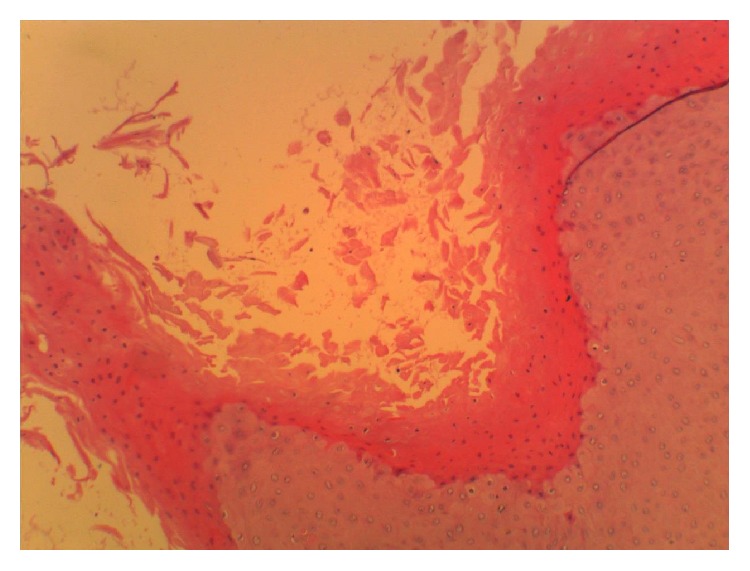
Cholesteatoma.

Incision and drainage of the right mastoid abscess with modified radical mastoidectomy were performed and revealed that a large cholesteatoma sac occupied the mastoid cavity and extended to the middle ear cleft. The histopathology results were consistent with cholesteatoma (Figure 3).

**Figure 4:**
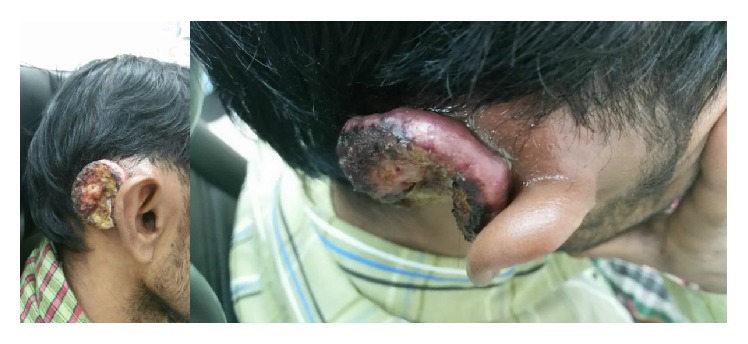
A 4 cm by 5 cm right postauricular ulcer with raised edges and a necrotic centre.

Postoperatively, the patient developed right postauricular wound dehiscence. The patient was lost to follow-up for seven months and later presented with 4 cm by 5 cm right postauricular ulcer with raised edges and a necrotic centre (base) (Figure 4).

**Figure 5:**
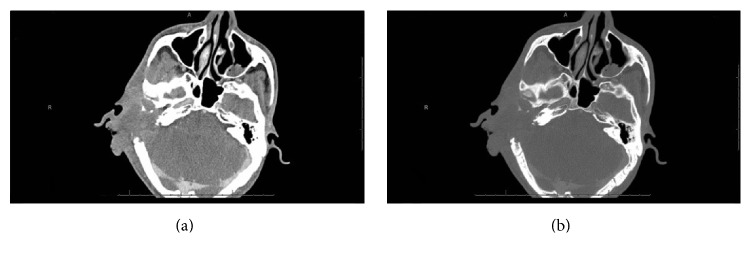
CT scan of the temporal bone shows that, compared to previous images, the soft tissue component was increased, causing further destruction of the middle and inner ear and a right periauricular soft tissue mass lesion with an ill-defined border.

Computerized axial tomography showed a right periauricular soft tissue mass with an ill-defined border (Figure 5).

**Figure 6:**
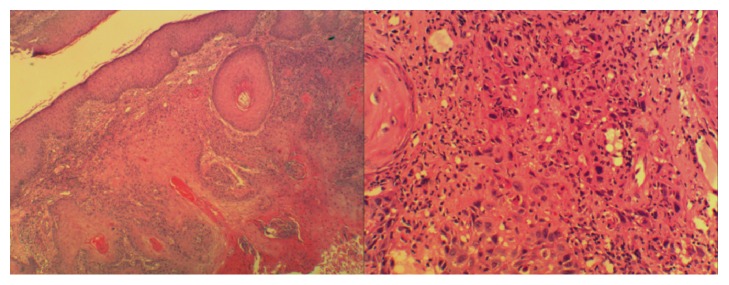
Section shows proliferative squamous cells invading the underlying stroma. The cells are hyperchromatic with a high N/C ratio and atypical mitosis.

Biopsies were taken from the edges and centre of the lesion. The biopsy from the edges showed moderately differentiated squamous cell carcinoma, and those from the centre showed dysplasia with keratinous material (Figure 6).

As the patient after diagnosis chose to return to his home country, no definite treatment was given to him.

## 3. Discussion

Marjolin's ulcer is a rare and often aggressive cutaneous malignancy that develops in previously traumatized or chronically inflamed skin, particularly after burns [7].

In the first century, Aurelius Cornelius Celsus was the first to report the development of a tumour in old burn scars and chronic nonhealing wounds. In 1828, Jean Nicholas Marjolin, a French surgeon, described a phenomenon that involved the formation of ulcerations within a burn scar and coined the term “ulcere cancroide”; however, the description did not say that the ulcers were malignant [13]. In 1838, Dupuytren observed that de novo malignancy could arise in chronic wounds; he observed this phenomenon in a Belgian man who was treated for a cancer that developed from a scar sustained from a sulphuric acid burn [14]. The name “Marjolin's ulcer” was first used by Da Costa in 1903, when he defined an ulcer arising from burn scars as Marjolin's ulcer [15].

Squamous cell carcinoma is the most common histological type among these wounds, followed by basal cell carcinoma, sarcoma, and melanoma [2, 10, 11, 16]. The male-to-female ratio increases with increasing patient age over 50 years [10, 11, 16].

Various theories have been proposed to explain the pathogenesis of the malignant transformation of these wounds, but none has provided a full explanation. The toxins theory, which proposes that the chronic inflammatory processes that lead to tissue damage produce toxins that may be carcinogenic, was proposed by Treves and Pack [11]. Virchow's theory of chronic irritation explains that, with chronic irritation and repeated tissue injury, the epithelium becomes less stable, loses contact inhibition, and undergoes malignant change. Other proposed theories include epithelial element implantation (Ribet's theory), the cocarcinogenic theory (Friedwald and Rose), and the immunologically privileged site theory. Castillo and Goldsmith suggested that the poor lymphatic flow in scar tissue impairs immunosurveillance, making it difficult for the body to mount an effective antigen-antibody response to protooncogens or tumours within scars. Hereditary theory and the environmental and genetic interaction theory seek to explain the evolution of acute Marjolin's ulcers by suggesting that genetic differences make the individual more susceptible to environmental insults, resulting in a short latency period.

As none of the above theories fully explain the evolution of Marjolin's ulcer, some studies have proposed a multifactorial theory consisting of various combinations of the current theories [3, 17, 18].

Latency has been described as the time between the primary pathology and the confirmation of a pathologic diagnosis of Marjolin's ulcer. The reported latency period for the development of malignancy is between 11 and 75 years [18]. Marjolin's ulcer can be classified as acute or chronic. In acute ulcers, the malignant degeneration occurs within 12 months; in the more common chronic ulcers, the degeneration occurs after 12 months.

When acute, the ulcer is most often basal cell carcinoma and is associated with a more superficial burn scar. However, acute malignant transformations to SCC do occur [5, 10, 19]. Many cases of acute transformation, ranging from weeks [18, 20] to months [7, 14, 21], have been reported in the literature.

Regarding the age of the patient and the burn scar, patient age is inversely proportional to the interval to the formation of cancer. The younger the patient is, the more likely he or she is to have a latency period of less than 1 year; older patients are increasingly likely to have a latency period greater than 1 year [10, 22, 23].

Marjolin's ulcer tends to be more aggressive than other types of skin cancer and has a higher rate of regional metastases [10]. However, head and neck lesions are associated with better survival, as are lesions of the upper extremities. Other factor associated with better survival include a latency to malignancy of less than 5 years, ulcers caused by burns, chronic osteomyelitis, a tumour size less than 2 cm, and ulcers less than 4 mm in thickness [3, 11].

Early diagnosis is essential. A high index of suspicion should be considered in the presence of chronic ulcers persisting longer than 3 months; rolled or everted wound margins; foul-smelling discharge; and an increase in pain, ulcer size, or bleeding [7, 9, 18, 22, 24]. Biopsy of suspicious lesions for histopathology remains the gold standard for diagnosis [24]. Many studies have recommended biopsies of multiple areas, such as the centre and margins [19], at appropriate depths [11].

Treatment of Marjolin's ulcer is quite varied. To prevent wound degeneration into squamous cell carcinoma, it is imperative to provide early and definitive wound coverage after burns and other traumatic injuries. Leaving large wounds to heal by secondary intention creates the potential for a chronic nonhealing ulcer and the ideal conditions for development of a Marjolin's ulcer. Wide local excision and subsequent skin grafting appear to be the standard of care for most authors [8]. MU is more aggressive than primary skin tumours; therefore nodal assessment and wide surgical excision are recommended [1].

## 4. Conclusions

Marjolin's ulcer is most commonly observed after postburn scars, but it may be observed after any type of scars, as our patient developed an SCC with a postsurgical scar. Early diagnosis is essential, and a biopsy should be performed on any nonhealing wound or chronic wound that undergoes a sudden change. Tissue samples should be taken from both the centre and the margins of the wound.

## Additional Points

*Summary.* We presented a case of a 30-year-old man who developed Marjolin's ulcer acutely in a right postauricular scar after mastoidectomy and the incision and drainage of a mastoid abscess. To the best of our knowledge, this report is the first to describe a Marjolin's ulcer in a postauricular surgical scar. Early diagnosis is essential, and a biopsy should be performed on any nonhealing wound or chronic wound that undergoes a sudden change.
